# Do Adolescent Exposure to Cannabinoids and Early Adverse Experience Interact to Increase the Risk of Psychiatric Disorders: Evidence from Rodent Models

**DOI:** 10.3390/ijms22020730

**Published:** 2021-01-13

**Authors:** Anna Portugalov, Irit Akirav

**Affiliations:** 1 Department of Psychology, School of Psychological Sciences, University of Haifa, 3498838 Haifa, Israel; anuta89.ap@gmail.com; 2 The Integrated Brain and Behavior Research Center (IBBR), University of Haifa, 3498838 Haifa, Israel

**Keywords:** cannabinoids, cannabis, adolescence, early life stress, depression, anxiety, rodent models

## Abstract

There have been growing concerns about the protracted effects of cannabis use in adolescents on emotion and cognition outcomes, motivated by evidence of growing cannabis use in adolescents, evidence linking cannabis use to various psychiatric disorders, and the increasingly perceived notion that cannabis is harmless. At the same time, studies suggest that cannabinoids may have therapeutic potential against the impacts of stress on the brain and behavior, and that young people sometimes use cannabinoids to alleviate feelings of depression and anxiety (i.e., “self-medication”). Exposure to early adverse life events may predispose individuals to developing psychopathology in adulthood, leading researchers to study the causality between early life factors and cognitive and emotional outcomes in rodent models and to probe the underlying mechanisms. In this review, we aim to better understand the long-term effects of cannabinoids administered in sensitive developmental periods (mainly adolescence) in rodent models of early life stress. We suggest that the effects of cannabinoids on emotional and cognitive function may vary between different sensitive developmental periods. This could potentially affect decisions regarding the use of cannabinoids in clinical settings during the early stages of development and could raise questions regarding educating the public as to potential risks associated with cannabis use.

## 1. Introduction

Exposure to early adverse life events is a major risk factor for developing psychiatric disorders such as depression and posttraumatic stress disorder (PTSD) in adulthood [1,2,3]. Although exposure to early life stress (ELS) has detrimental immediate effects on the activation of the stress system (and other systems), there are long-term effects that emerge at later stages of development, either in adolescence or later [4,5]. 

There is growing interest in the use of cannabis and cannabinoids to prevent and treat psychiatric disorders [6], and self-medication with cannabis is often related to the treatment of psychiatric conditions [7,8,9]. Specifically, there is an increasing body of clinical [10,11,12,13] and preclinical [14,15,16,17,18,19] literature indicating that enhancing endocannabinoid (ECB) signaling protects against the effects of stress and ameliorates stress-induced alterations in depression and anxiety-related disorders [20,21]. Conversely, disruption of ECB signaling worsens the neurobehavioral and hormonal responses to stress, impairs appropriate termination of stress responses, compromises adaptation to stress, and promotes structural changes in the brain associated with mood and anxiety disorders [22,23,24].

However, exposure to cannabinoids during adolescence has been suggested to have protracted long-term detrimental effects on emotional and cognitive function, possibly contributing to the development of the pathological effects of chronic stress and to the development of psychiatric disorders [25]. This is an important issue, as there is a high prevalence of cannabis use among adolescents [26] and cannabis is increasingly viewed as harmless by both adolescents and adults [27].

Given the susceptibility to developing psychiatric symptoms following early adverse experiences and the increase in cannabis use among adolescents worldwide, the aim of this review is to characterize the sustained effects of ELS and cannabinoid exposure on emotional and cognitive well-being in adulthood based on rodent models. Cannabinoids administered during sensitive developmental periods (primarily adolescence) to individuals exposed to adverse events early in life may restore the long-term detrimental effects of ELS on emotion and cognition, or may have an additive effect with ELS to exacerbate the symptoms. As we will describe later, cannabinoids may also have no additive effect to that of ELS or can have opposing effects in the same experimental setting.

In particular, it is interesting to examine whether vulnerable individuals that have been previously exposed to adverse events are at higher risk of developing a disorder following cannabis exposure. A better understanding of the long-term effects of cannabinoids used in sensitive developmental periods in vulnerable individuals could potentially affect the decision to administer cannabinoids during early stages of development in clinical settings and could encourage regulators to acknowledge the potential opportunities and risks that cannabis and cannabis-derived compounds may have.

It should be noted that although the association between early life adversity and mood and anxiety disorders is widely accepted, many people that are exposed to childhood adversity do not develop mental disorders [28]. Humans, as well as rodents, may display resilience or an adaptive response to stress [29]. On the other hand, mental disorders also develop in many people that were not necessarily abused or exposed to severe stress in early life.

Here, we will give a brief overview of the early stress models in rodents and their long-term effects [30,31,32], as well as a short overview of the ECB system and the developmental axis. Then, we will focus on rodent studies that examine the long-term effects of exposure to ELS preweaning and to cannabinoids administered during sensitive developmental periods. We will also describe sex differences in the responses to ELS and cannabinoid exposure in adolescence and discuss the relevant human studies.

## 2. Long-Term Effects of Early Stress Models in Rodents

Traumatic experiences (such as abuse, neglect, loss of a parent) during early developmental periods might be associated with psychopathology (such as depression, anxiety disorders, schizophrenia) and altered neuroendocrine function and disruption of the hypothalamic–pituitary–adrenal (HPA) axis later in life [31,33,34]. Depression and anxiety are uniquely human, and the validity of ELS models in rodents for depression is limited [32]; yet rodent models enable to study the causality between adverse early life experiences and behavioral and cognitive outcomes, as many symptoms of these disorders can be modeled [31,32]. Furthermore, animal studies facilitate the investigation of the pathophysiological and neuroendocrine pathways of psychopathologies of depression- and anxiety-related disorders [31,32,33,34]. This knowledge may lead to identification of new potential targets for novel pharmacotherapies and prevention strategies in humans.

In rodents, “early life” can be divided into three periods of time: the prenatal, postnatal (until postnatal day (P) 21, preweaning), and early adolescence (P21–30) periods. In the current review, we will focus on adverse experiences during the postnatal, preweaning period (until P21) in rodent models. The models of ELS can be subdivided in two main groups: pharmacological models (internal stressors) and interventions in mother–pup interactions (external stressors).

### 2.1. Pharmacological Models

Pharmacological models of ELS manipulate internal mediators, such as hormones, neurotransmitters, and inflammatory mediators [32]. These models target the HPA axis and disrupt the development of the stress system [32]. An example of such a model is postnatal treatment with dexamethasone—Wistar rats treated with dexamethasone on P3–6 exhibited anxiety-like behaviors in an open field test and an elevated plus maze (EPM) test in adulthood [35]. Another model for pharmacological ELS is postnatal lipopolysaccharide (LPS), which mimics bacterial infections in human infants. Recent studies have demonstrated the interactions between postnatal inflammatory insults with live bacteria and behavioral and neuroendocrine changes in adulthood [36,37,38,39]. LPS-treated rats at P3–5 demonstrated anxiety-like behavior, spending more time in the closed arms of the EPM and exhibiting less exploratory behavior in the hole board apparatus in adulthood [40]. These behavioral changes were associated with corticotropin-releasing hormone (CRH) receptor-1 (CRHR1) mRNA downregulation in the prefrontal cortex (PFC) and hypothalamus, as well as upregulation in the hippocampus, suggesting disruption of the HPA axis [40]. In another study, CRHR1 expression was downregulated in the CA1 and CA3 regions of the hippocampus and upregulated in the hypothalamus in mice treated with LPS on P3 and P5 [41].

### 2.2. Mother–Pup Interaction Manipulations

Rodent models have shown the deleterious impact of inadequate maternal care during critical developmental periods on adult social behavior [42], cognition [43], gene expression [44], and brain function [45].

In the highly prevalent maternal separation (MS) model, pups are separated from the dam for distinct intervals of time [30,32,46]. In one study, Sprague–Dawley (SD) pups that underwent MS for 3 h a day during P1–14 spent more time in immobility in the forced swim test (FST) (i.e., learned helplessness, suggesting depression-like behavior) and more time in the closed arms in the EPM in adulthood (i.e., increased anxiety-like behavior), and also showed a decrease in serotonin levels in the hippocampus, which is associated with depression [47]. In another study, adult rats that had undergone MS for 6 h daily during P1–13 travelled less distance in the open field test, demonstrated a higher ratio of immobility in the FST, and consumed less sucrose compared to the control group, indicating an anhedonic, depression-like response [48]. Additionally, Bai et al. [48] demonstrated epigenetic changes in maternally separated rats, exhibiting low expression of brain-derived neurotrophic factor (BDNF) mRNA, which was positively correlated with the distance traveled in the open field test, suggesting hypolocomotion and high microRNA (miR)-16 expression in the hippocampus, which were negatively correlated with the sucrose preference rate, suggesting anhedonia [48]. The microRNA are small non-coding RNA molecules that function in RNA silencing and posttranscriptional regulation of gene expression, and have been demonstrated to play a role in the maladaptive processes associated with ELS and depression [49].

An associated model is the maternal deprivation (MD) model, in which there is a single separation of the pups from the dam for 24 h [30,32,46,50]. Ample evidence has demonstrated the impact of a single prolonged episode of separation from the dam at critical developmental points [31]. Roceri et al. [51] found a reduction in the expression of brain-derived neurotrophic factor (BDNF) and N-methyl-D-aspartate receptor (NMDAr) subunits in the hippocampus of rats exposed to 24 h of MD on P9. Ellenbroek and Cools [52] demonstrated that MD on P9, but not on P13, led to disruption of prepulse inhibition (PPI, disrupted sensory gating) in adulthood. Furthermore, rats exposed to 24 h of MD on P9 exhibited a significant increase in immobility and a significant decrease in climbing and swimming in the FST in late adolescence [53]. These behavioral changes were associated with an increase in excitability and burst activity in the lateral habenula, a depression-like phenotype [54].

The limited bedding and nesting (LBN) model was developed to specifically model neglect, as it is not clear whether the MS and MD manipulations model neglect, abuse, or both, since the observed effects could be due to the altered maternal behavior towards the pups that is observed after the separation [55]. In the limited bedding and nesting paradigm or neglectful mother paradigm [42,43,56,57,58,59,60,61,62], the mother handles her pups roughly when provided with insufficient bedding for nest building. The LB environment decreases the ability of the mother to construct a nest, which results in frequent nest building, spending more time away from the nest, rough handling, and stepping on pups. Ivy and colleagues [61] found that LB influenced the quality of the dams’ care. They observed a decrease in licking and grooming behavior, and more frequent leaving of the pups. Furthermore, the dams with restricted nesting material exhibited anxiety-like behavior in the open field test, which was accompanied by increased levels of plasma corticosterone and adrenal weights (indicators of chronic stress; [61]). These maternal behaviors may lead to continuous disturbances in the pups, which in turn increase the risk of susceptibility to affective disorders later in life [42,56]. Avishai-Eliner et al. [56] found that P9 pups immediately after 7 days of LB had reduced expression of CRH mRNA and glucocorticoid mRNA in the paraventricular nucleus and frontal cortex. McGgowan et al. [44] also suggested that maternal care is associated with long-term effects on epigenetic programming, as adult rats with low maternal care mothers demonstrated epigenetic changes in promotors, exons, and gene ends associated with lower transcriptional activity compared to rats with high maternal care mothers.

As the literature about the deleterious short- and long-term effects of ELS models on brain and behavior is beyond the scope of this review, we will focus here on the interaction between ELS and exposure to cannabinoids later in life.

## 3. The Endocannabinoid (ECB) System

The ECB system is a neuromodulatory lipid system, which includes the cannabinoid receptors CB1 and CB2; two major endogenous ligands, namely N-arachidonyl ethanolamine (anandamide; AEA) and 2-arachidonoyl glycerol (2-AG); and enzymes that catalyze synthesis and degradation, namely 2 monoacylglycerol lipase (MAGL) for 2-AG and fatty acid amide hydrolase (FAAH) for AEA [63]. CB1 receptors (CB_1_R) are the most abundant G-protein-coupled receptors in the mammalian brain, but are also present at much lower concentrations in a variety of peripheral tissues and cells [64]. CB2 receptors (CB_2_R) are expressed primarily in cells of the immune and hematopoietic systems, but are also present in the brain. The endogenous ligands AEA and 2-AG are not stored but rather are produced on demand in response to a depolarization-induced rise in intracellular calcium or activation of various metabotropic receptors [64].

Generally, disruption of ECB signaling leads to hormonal and neurobehavioral changes, inappropriate termination of stress response, and structural brain changes related to mood and anxiety disorders [24,65]. The ECB system also has a prominent role during the developmental process, guiding axons and circuit formation and regulating synaptic transmission [66,67,68]. The ECB system is important in the regulation of stress and is also modulated by exposure to stress [69,70,71,72]. Furthermore, it is an important modulator of emotional behavior and mood [73,74], and is involved in brain reward processes and drug addiction [75,76].

Importantly, the ECB system has been recently suggested to be involved in the etiology of depression and anxiety-related disorders. Enhancement of ECB signaling may play an important role in alleviation of depressive- and anxiety-like symptoms [14,17,18,19]. Evidence of cannabis use for alleviating symptoms of depression in humans [77] is backed by animal research [15,78,79]. Rodent studies suggest beneficial effects of ECB signaling enhancers such as the FAAH inhibitor URB597 (URB; FAAH degrades AEA), the CB1/2 receptor agonist WIN55,212-2 (WIN), and cannabidiol (CBD; a cannabis sativa constituent) in rodent models for depression [15,80,81,82], anxiety, and PTSD [16,83,84,85,86]. Although there is support for a therapeutic effect of cannabinoids in humans [10,11,12,87,88,89,90], most clinical studies to date have significant limitations (e.g., small samples, low quality), complicating the establishment of recommendations for clinical cannabinoid use.

While cannabis use is associated with mood augmentation and stress relief, it can also induce dysphoric responses, such as heightened anxiety [91]. In this regard, rodent models demonstrate dose-dependent effects of CB_1_R direct and indirect agonists, suggesting that low to moderate doses of CB_1_R agonists exhibit anxiolytic effects [92].

Recent studies suggest that the ECB system is vulnerable to early life adverse experiences [93,94,95,96], and its impairment can lead to psychopathologies later in life [97,98,99,100,101]. Hill and colleagues [93] demonstrated persistent and instantaneous changes in the ECB system measured after exposure to MS (P2–12), as well as under basal conditions in juvenile (P14), adolescent (P40), and adult (P70) rats. AEA content was found to increase from P2 into adulthood in a linear manner in the hippocampus, amygdala, and PFC. 2-AG showed a similar pattern in the hippocampus. In the amygdala and PFC, 2-AG increased during the juvenile period (P12–14). CB_1_R receptor densities were downregulated later in life following exposure to MS. Hence, MS modulates ECB levels in neonates, resulting in deficits in ECB function, particularly within the hippocampus, in adulthood. Amancio-Belmont et al. [97] also observed a reduction in CB1R expression in the PFC and an increase of CB1R expression in the nucleus accumbens in adult rats that were exposed to MS at P2–P15. These changes in CB1R expression due to MS were associated with higher alcohol intake in adulthood [97], suggesting that ELS can lead to persistent changes in the ECB system, which in turn can be the basis for psychopathologies later in life.

## 4. The Developmental Axis

Development represents a crucial period for shaping adult behavior, and may impact disease vulnerability later in life. An increasing body of evidence suggests that environmental factors, particularly during the developmental period, account for a significant proportion of susceptibility to psychiatric conditions [102]. In this regard, animal models can complement human studies and constitute a valuable tool for the investigation of critical periods in human brain development. Marco et al. [102] suggested three critical periods during development—the prenatal period, through to the first years of life, and then until adolescence. In this review, we will focus on the exposure to stress in infancy and exposure to cannabinoids during adolescence in rodent models.

Infancy in rodents refers to the first three weeks after parturition [103]. The first two weeks are particularly critical in young rodents (P4–14 in rats and P1–14 in mice; [104,105,106], and this developmental period is named the “stress hypo-responsive period” (SHPR), characterized by low basal corticosterone secretion and the inability of stressors to elicit a corticosterone response [104,105,106,107,108]. This period is critical for intact development of the HPA axis, and ample evidence suggests that different aspects of the dam’s behavior appear to regulate different features of the HPA system [107]. Thus, most prevalent animal models of ELS are performed during this period.

Another critical period in the developmental process is adolescence, which covers the complete time span between childhood and adulthood [109]. Adolescence is a period of neural imbalance caused by the relatively early maturation of subcortical brain areas and the relatively delayed maturation of prefrontal control areas, with the result that in emotional situations, the more mature limbic and reward systems gain the “upper hand” over the still relatively immature prefrontal control system [110,111]. This happens in parallel to an increase in white and grey matter, together making adolescents more vulnerable to harmful environmental influences, e.g., drugs and cannabis use [109]. It is hard to define the time course of adolescence, with no single event signaling its onset or termination [109]. A widely used and conservative age range classifies rodents as adolescents at P28–P42, with weaning most prevalently occurring within P21–P25, which is designated as a juvenile period, and adulthood classified as beginning at P60 [109]. Here, we will depend on a less conservative classification system used for adolescence in rats, which includes three subgroups: a peri-adolescence or early adolescence period from P21 to P34, a mid-adolescence period from P34 to P44, and a late adolescence period from P45 to P59 [103].

## 5. Do Cannabinoids Exacerbate or Ameliorate the Long-Term Effects of ELS Exposure?

### 5.1. ELS and Cannabinoid Exposure at P10 (Infancy)

In several studies, the treatment with cannabinoids was performed immediately after the ELS model. In one study, male and female rats were exposed to MD for 24 h on P9 and to a single injection of the CB1/CB2 receptor agonist WIN (0.1 mg/kg, i.p.) on P10 [112]. MD decreased activity in the hole board test and EPM test in males, suggesting increased anxiety-like behavior, increased immobility time in the FST in males and females, and decreased corticosterone levels in females. WIN by itself decreased activity in the hole board test and had an anxiogenic-like effect in the EPM in males; in both males and females, WIN induced depression-like behavior and increased corticosterone levels. Interestingly, WIN reversed the MD-induced decrease in activity in the hole board test and EPM in males, but exacerbated the effects of MD on corticosterone levels in females [112].

In another study by McLaughlin et al. [113], rats were exposed to LB at P2–9 and on P10 intraperitoneally (i.p.) injected with the FAAH inhibitor URB (0.3 mg/kg) 90 min prior to immobilization stress. URB decreased stress-induced corticosterone release in pups exposed to ELS, but not in no-ELS pups exposed to the immobilization challenge. The authors suggested that enhancing AEA mitigates stress-induced alterations in glucocorticoid secretion preferentially in pups subjected to ELS. Further studies are needed to establish the effects of cannabinoid exposure in infancy in ELS models.

### 5.2. ELS and Cannabinoid Exposure during Mid-Adolescence

Findings regarding cannabinoids administered during mid-adolescence reveal conflicting results—studies show therapeutic long-term effects of cannabinoids administered during adolescence in rodents exposed to ELS [114,115], but mounting evidence in rodents also suggests that exposure to cannabinoids during adolescence might act as a risk factor for the occurrence of psychiatric disorders later in life [25,116].

Several studies suggest that exposure to cannabinoids during adolescence can be therapeutic or at least not harmful in a dose-dependent manner. Macri and Laviola [117] tested CD-1 mice of both sexes that underwent MD on P12 and were administered the CB1/2 agonist WIN (0, 0.5, or 2 mg/kg, i.p.) for 3 days during adolescence (P35–37). MD reduced the expected interest in socio-sexual interaction with peers during adolescence. When MD-untreated mice were tested in adulthood in the FST, the time to reach a passive floating posture was significantly reduced, suggesting learned helplessness. A low dose of WIN (0.5 mg/kg) administered during adolescence did not change immobility levels in MD mice, but restored the social behavior. Altogether, their findings suggest that WIN increased the ability of adult mice to cope with a stressful environmental challenge [117].

A similar pattern emerged when rats that were exposed to MD on P9 were treated with different doses of the FAAH inhibitor URB (0.1 or 0.5 mg/kg, i.p.) for six days during adolescence (P31–43), during which time they were also tested in the intolerance-to-delay task (an impulsivity test) [114]. MD increased impulsivity and locomotor response to novelty when compared to non-MD rats. The low dose of URB (0.1 mg/kg) effectively decreased impulsive behavior specifically in MD subjects, suggesting increased self-control behavior, and increased the levels of N-acetyl-aspartate (NAA) and creatine in the hippocampus only in MD subjects, suggesting an augmentation of neurogenesis in the hippocampus. The high dose of URB (0.5 mg/kg) did not affect behavior but downregulated glutamate levels, suggesting a schizophrenia-like phenotype.

Doenni et al. [118] also demonstrated a therapeutic effect of an FAAH inhibitor in ELS rats. Male and female rats were exposed to LPS injection at P14 and tested for social behavior in adolescence (P40). LPS-injected rats exhibited decreased social behavior, which correlated with decreased CB1 binding, increased AEA levels, and increased FAAH activity in the amygdala. Oral administration of the FAAH inhibitor PF-04457845 (1 mg/kg) on P40, four hours before social testing, reversed the social impairment in LPS-treated rats. Additionally, only in females did infusion of PF-04457845 (10 ng) directly into the basolateral amygdala (BLA) increase social behavior compared to the non-LPS-treated base levels [118].

The synthetic delta-9-tetrahydrocannabinol (THC) dronabinol administered during adolescence has also been shown to reverse some deleterious effects of ELS. Morel et al. [115] exposed male rats to MS for 3 h a day during P1–14, and for two weeks during adolescence (P35–49) administered the CB1/CB2 agonist dronabinol (5 mg/kg or 10 mg/kg i.p.); to mimic the intermittent and escalating use seen in teenagers, the administration was performed with days of abstention and increased doses until the dose 10 mg/kg. Both MS and chronic dronabinol treatment resulted in hypersensitivity to the reward effect of morphine in the conditioned place preference paradigm. However, chronic dronabinol treatment in MS rats suppressed sensitivity to the reward effect of morphine in the same test, suggesting that THC ameliorated the deficits of exposure to adverse experiences early in life [115].

Similar findings were obtained by Zamberletti et al. [119], who treated male and female rats exposed to MD on P9 with increased doses of THC (2.5, 5, and 10 mg/kg, i.p.) twice a day for ten days during adolescence (P37–47), then tested the rats in adulthood. In females, THC reversed aggressive behavior in MD rats in the social interaction test. Furthermore, in both sexes, THC counteracted the increase in NMDAr density and the reduction in D2 dopaminergic receptor density in the caudate–putamen complex caused by ELS. However, THC also impaired the performance of non-MD exposed females in the social recognition test and increased their time of immobility in the FST. Exposure to THC in MD animals increased the time of immobility in the FST in males and resulted in downregulation and desensitization of CB_1_R in both sexes [119]. Hence, there are strong indications of beneficial effects of ECB system activation following ELS; however, these findings also suggest a more complex picture of the effects of cannabinoids on the brain and behavior, as there are indications of non-beneficial or even harmful effects within the same experimental setting.

Other studies show harmful effects of exposure to cannabinoids during adolescence following ELS. Chronic treatment with the cannabinoid agonist CP-55,940 (CP; 0.4 mg/kg, i.p.) did not reverse anxiety-like behavior in MD male rats in the EPM test [120]. The drug in non-MD conditions induced a disruption in PPI in females and augmented adrenocortical responsiveness to the PPI test in males. Both MD and chronic treatment with CP reduced plasma levels of leptin, while in females only MD caused the same reduction. Additionally, in males, CP increased the levels of corticosterone and the adrenocorticotropic hormone (ACTH) [120]. The same group also examined CB_1_R density, glial fibrillary acidic protein-positive (GFAP+) cells, and BDNF expression in the hippocampus of the same animals [100]. In males, MD and CP separately each induced a decrease in CB_1_R density in the dentate gyrus and CA1 and an increase in GFAP+ cells in the dentate gyrus. However, CP reversed the reduction in CB_1_R density in the dentate gyrus and the increase in GFAP+ cells in MD males. An increase in GFAP+ cells may reflect changes in astrocyte reactivity, which is associated with the development of reward effects and drug dependence [100,121]. In females, exposure to CP during adolescence reduced the expression of BDNF in the CA1 and CA3, whereas MD elevated the expression of BDNF in the dentate gyrus [100].

Our group also found a mixture of beneficial and detrimental long-term effects of the CB1/CB2 agonist WIN (1.2 mg/kg) administered during adolescence (P30–45) to male rats that were exposed to LB at P7–14 [43]. When compared to ELS rats treated with vehicle during adolescence, ELS rats treated with WIN demonstrated impaired performance in spatial recognition and social recognition memory tasks in adulthood (P75). However, ELS WIN-treated males also demonstrated less anxiety-like behavior in an open field [43].

We recently found the FAAH inhibitor URB (0.4 mg/kg, i.p.) to have only deleterious effects on male and female rats exposed to LB at P7–14 when administered for 2 weeks at P30–45 [122]. When tested in adulthood (P75), ELS-exposed rats exhibited impaired performance in the social preference and social recognition tests, demonstrated increased immobility in the FST, and anxiety-like behavior in the open field test. Chronic treatment with URB for 2 weeks during adolescence did not prevent the detrimental effects of ELS in either sex. Furthermore, exposure to URB without ELS resulted in downregulation of CB_1_R in the PFC and CA1 and downregulation of glucocorticoid receptors (GR) in the PFC and BLA in males. In females, ELS downregulated the expression of GRs in the CA1 and BLA and downregulated BLA-CB_1_R; likewise, in females, URB downregulated the expression of GRs in the CA1 and downregulated BLA-CB_1_R. Interestingly, when WIN or URB was administered to ELS-exposed rats postadolescence (P45–60), ELS-induced deficits in behavior and brain function were reversed [43,123], suggesting a therapeutic potential for cannabinoids in the postadolescence period. This will be elaborated in the next section.

For a summary of the long-term effects of ELS and cannabinoids administered during mid-adolescence, see Table 1.

### 5.3. ELS and Cannabinoid Exposure during Late Adolescence

The findings regarding exposure to cannabinoids during late adolescence are more consistent with a beneficial outcome. We examined male and female rats exposed to LB during P7–14, injected with WIN (1.2 mg/kg, i.p.) for 2 weeks during late adolescence (P45–60), and tested at P90 [43]. ELS males and females in adulthood demonstrated impaired performance in short-term memory in the spatial location and social recognition tasks; males were also impaired in the novel object recognition task. WIN administered during late adolescence prevented these ELS-induced deficits and reduced anxiety levels in an open field test. WIN was also shown to normalize the ELS-induced upregulation of PFC-GRs and CA1-CB_1_R in females. In males, WIN normalized the ELS-induced upregulation of PFC-GR and downregulation of BLA-CB_1_R. As mentioned in the last section, when WIN was administered during mid-adolescence (P30–45) to ELS male rats, the effects of LB were not restored [43].

A similar beneficial effect was observed when the FAAH inhibitor URB (0.4 mg/kg, i.p.) was injected to LB rats for two weeks during late adolescence (P45–60) [122]. Compared to ELS vehicle-treated rats, adult ELS male and female rats that had been treated with URB during late adolescence showed normal levels of social preference, intact social recognition, and normalization of the ELS-induced increase in immobility in the FST; males treated with URB during late adolescence also showed normalization of the ELS-induced increase in freezing in an open field test. Nevertheless, female rats treated with URB during late adolescence still demonstrated increased freezing in the open field test.

A beneficial effect was also observed in a recent study [123] of male and female rats exposed to LB during P7–14 and treated for 2 weeks at P45–60 with URB (0.4 mg/kg, i.p.) or the MAGL inhibitor JZL184 (JZL; 2 mg/kg, i.p.). In both sexes, ELS resulted in impaired performance in the social preference and social recognition tests, higher immobility in the FST, reduced activity of the enzyme MAGL in the ventral subiculum, and reduced long-term potentiation (LTP) in the ventral subiculum–nucleus accumbens pathway. Additionally, in males, LB reduced BDNF expression in the nucleus accumbens and ventral subiculum. In females, ELS elevated BDNF expression in the ventral subiculum and reduced the activity of MAGL in the nucleus accumbens [123]. Chronic treatment with URB or JZL improved the performance in the social preference and social recognition tests and reduced passive coping in the FST in both sexes. Moreover, treatment with URB normalized BDNF expression in the ventral subiculum in males and females. Treatment with JZL normalized BDNF expression in the ventral subiculum in males and normalized MAGL activity in the nucleus accumbens in females [123]. Taken together, these last two studies suggest a strong therapeutic potential of URB and JZL administered during late adolescence to male and female rats exposed to ELS. To summarize, these studies suggest that enhancing ECB signaling may have deleterious or ameliorating effects on behavior, depending on whether the developmental time window of treatment is in mid- or late adolescence.

For a summary of the long-term effects of ELS and cannabinoids administered during late adolescence, see Table 2.

### 5.4. Direct Versus Indirect Agonists of the Endocannabinoid System

Manipulation of the ECB system through indirect agonists that increase concentrations of AEA and 2-AG has become a major focus of recent research as a more efficient therapeutic target for depression and anxiety-like disorders than direct agonists of CB_1_R [124]. A PET study suggested that direct activation of CB_1_R leads to downregulation of ECB signaling and can exacerbate depression- and anxiety-like symptoms [125]. This downregulation was also demonstrated in animal models [122]. Moreover, direct activation of CB_1_R can result in negative side effects, such as addiction, catalepsy, and hypothermia [126,127]. On the contrary, indirect FAAH inhibitors have a prominent selectivity for FAAH, with no activity on other ECB components (i.e., CB_1_R, CB_2_R, MAGL), and thus have less harmful side effects [126]. Nevertheless, other factors (e.g., dosing, developmental phase) are also critical in determining the outcome of treatment, as it was demonstrated that during adolescence, administration of indirect agonists such as URB may result in a negative outcome [43,122], while direct agonists of CB_1_R, such as CP and THC, can reverse some deleterious effects of ELS [100,119].

## 6. Sex Differences

As there are widely-known sex differences in the stress response system and the ECB system [128], it is only reasonable that there are sex differences observed in behavior following exposure to ELS and cannabinoids. These differences are already observed in infancy. For example, MD or WIN injection on P10 decreased activity in the hole board test and EPM only in males, while MD for 24 h on P9 decreased corticosterone levels only in females [112]. Moreover, WIN reversed the MD-induced decrease in activity in the hole board test and EPM in males but exacerbated the effects of MD on corticosterone levels in females [112]. These differences are presumably due to the organizational effects of perinatal androgens during the crucial period of brain sexual differentiation [129].

During adolescence, the sex differences are more prominent. Macri and Laviola [117] found that MD reduced the time to reach a passive floating posture and a high dose of WIN (2 mg/kg) decreased locomotion more markedly in male mice than in females, while in the FST, males took more time to exhibit the floating posture (i.e., immobility) compared to females in all groups. Furthermore, infusion of the FAAH inhibitor PF-04457845 (10 ng) to the BLA reversed LPS-induced social impairment only in females [118].

Zamberletti et al. [119] observed sex differences in the influence of THC during adolescence on behavior—THC impaired females’ performance in the social recognition test and increased their time of immobility in the FST. The impairment in the social task only in females may be in accordance with the fact that only among women was an association found between cannabis use and social anxiety disorder [130]. Exposure to THC in MD males increased the time of immobility in the FST [119]. Notably, this depressive-like behavior was exhibited only in the case of the dual MD and THC insult, but not in the case of either one on its own.

The agonist CP also induces sex-dependent effects. Exposure to CP during adolescence resulted in disrupted PPI only in females, while in males the result was an augmentation of adrenocortical responsiveness and an increase in the levels of corticosterone and ACTH [120]. Additionally, CP reduced plasma leptin only in males, while MD reduced plasma leptin in both sexes. Finally, Alteba et al. [122] demonstrated sex-dependent effects of ELS and URB on the expression of CB_1_R and GRs in the PFC–hippocampal–BLA circuit, as described in the previous section.

These sex differences in adolescence appear due to gonadal maturation during puberty and testicular and ovarian hormones, which act to facilitate expression of sex-typical behaviors in adulthood [131]. It has been suggested that the adolescent brain is reorganized a second time by gonadal steroid hormones secreted during puberty, building on and refining circuits that were sexually differentiated during early neural development [132].

Other studies found sex differences in the ECB system in postadolescence and adulthood. For example, adult male rats show higher levels of hippocampal CB_1_R expression than females [133]. In addition, the affinity of ligands for limbic forebrain CB_1_R is significantly lower in females than males [134]. These findings can explain the evidence that chronic treatment with CP (0.2 mg/kg) during postadolescence induced anxiety-like behaviors only in males but not in females [135]. Additionally, WIN administration during late adolescence prevented ELS-induced impairment in the novel object recognition task only in males [43], while WIN normalized the ELS-induced alterations in CB_1_R and GRs in the PFC–hippocampal–BLA circuit in a sex-dependent manner. Additionally, treatment with URB or JZL during late adolescence normalized ELS-induced alterations in BDNF expression and MAGL activity in the nucleus accumbens and ventral subiculum in a sex-dependent manner [123].

Women display a higher prevalence of mood and anxiety disorders than men [136]. However, some studies on rodents show that in females, exogenous challenges such as ELS produce no effect or a decrease in depression- and anxiety-like behavior [137,138,139,140]. One possible explanation is that the typical laboratory tests of anxiety- and depressive-like behavior are not good measures for behavior in females—these measures were developed in males and usually measure behavioral inhibition, whereas females showed variations in activity levels due to fluctuations in the estrous cycle [55].

## 7. Human Studies

Individuals exposed to stress early in life have been hypothesized to develop pathophysiological changes in the central nervous system that increase their vulnerability to stress later in life, predisposing them to developing psychopathologies [141]. Cannabis use during adolescence has been suggested as a risk factor for neuropsychiatric disorders [142,143,144,145,146,147,148] and adolescent-onset cannabis users demonstrated greater neuropsychological decline compared to adult-onset cannabis users [146,149,150,151,152].

However, cannabis is a prevalent coping tool for dealing with negative feelings and problems [153]. Feingold et al. [154] suggested that poorer outcomes of anxiety disorders among cannabis users stem mainly from differences in baseline factors and not cannabis use, and in particular to baseline differences in clinical factors. The reported attempts to reduce depressive- and anxiety-related clinical effects by using cannabis (i.e., “self-medication”) further complicate the association between cannabis use and outcomes of various psychiatric disorders.

Likewise, Ketcherside and Filbey [155] suggested that the association between ELS and cannabis use is mediated by depression—individuals exposed to ELS are prone to develop depression, and such individuals use cannabis in order to cope with the negative effects. Furthermore, it was found that experiencing more early life stressors was associated with more frequent cannabis use and more long-term problems from use [156]. Thus, ELS may be interpreted as a risk factor for cannabis use during adolescence, which in turn can serve as a second risk factor for development of psychopathology. In support of the self-medication hypothesis, i.e., using cannabis to alleviate feelings of depression and anxiety, Bujarski et al. [157] found that marijuana-using adolescents’ posttraumatic stress symptoms over the past two weeks significantly predicted coping motives for marijuana use—their motives were not associated with social or conformity reasons for use, but rather to stress relief.

Hence, research shows that young people sometimes use cannabinoids to alleviate feelings of depression and stress (“self-medicating”), when in fact using cannabis during adolescence can compound the problem [149,150,151,152]. The result may be that young depressed or anxious individuals that use cannabis may increase their chance of suffering a more severe mental health problem. This is further complicated by the fact that it is fairly difficult to study self-medication, as it does not happen in a controlled setting and the sources of the cannabis consumed are unregulated, making it hard to reach conclusions that can inform clinical practice on how to prescribe cannabis adequately. A recent meta-analysis argued that there is insufficient evidence to provide guidance on the use of cannabinoids for treating mental disorders within a regulatory framework [158].

High-quality clinical studies directly examining the effects of cannabinoids on treating mental disorders are needed.

## 8. Conclusions

This review demonstrates age-dependent long-term positive and negative psychological effects of cannabinoids in interactions with early adverse life experiences. Depending on a variety of factors such as sex, the type of cannabinoid administered, age at administration, and dosing, cannabinoids may either counteract or exacerbate adversity-caused conditions, and even sometimes counteract one condition while exacerbating another. The complicated picture emerging from these findings emphasizes that adolescence is a particularly sensitive period for exogenous cannabinoid administration, especially when in conjunction with previous adverse experiences. The results strengthen the existing concern that use of cannabis, whether prescribed or “self-medicated”, to treat psychological conditions or stress may put users, especially adolescents, at risk for developing mental health problems of greater severity. This study underscores the need for increased public awareness of the multipronged impacts of cannabis use, especially in adolescents, to challenge the widely-held view that cannabis is harmless, and for further research to inform clinical and regulatory decision-making.

## Figures and Tables

**Table 1:** Summary of rodent model studies of exposure to cannabinoids during mid-adolescence and its interaction with long-term effects of ELS.

Reference	Animals	ELS Effects	Cannabinoid Effects	Interaction Effects
[117]	CD-1 mice (both sexes)	MD on P12 Reduced interest in socio-sexual interaction with peers during adolescence (both sexes). Depressive-like behavior in the FST in adulthood (both sexes).	WIN on P35–37 (0.5 mg/kg, i.p.) Increased active coping in the FST (both sexes). WIN on P35–37 (2 mg/kg, i.p.) Reduced social investigation and locomotor activity (both sexes).	WIN on P35–37 (2 mg/kg, i.p.) Reduced locomotor activity (both sexes).
[114]	Wistar rats (male)	MD on P9 Increased impulsivity and locomotor response to novelty in the intolerance-to-delay test (male).	URB on P31–43 (0.1 or 0.5 mg/kg, i.p.) No effects reported.	URB on P31–43 (0.1 mg/kg, i.p.) Decreased impulsivity (male). Increased NAA and creatine levels in the hippocampus (male). URB on P31–43 (0.5 mg/kg, i.p.) Downregulated glutamate levels.
[118]	SD rats (both sexes)	LPS on P14 Decreased social behavior during adolescence (both sexes). Decreased CB_1_R binding (both sexes). Increased AEA levels and FAAH activity in the amygdala (both sexes).	PF-04457845 on P40 (1 mg/kg, orally) No effects reported.	PF-04457845 on P40 (1 mg/kg, orally) Restored social behavior (both sexes). PF-04457845 on P40 (10 ng, intra-BLA) Restored social behavior (females).
[115]	Long–Evans rats (both sexes)	MS on P1–14 Hypersensitivity to the reward effect of morphine in the place preference paradigm (both sexes).	Dronabinol on P35–49 (5 and 10 mg/kg, i.p.) Hypersensitivity to the reward effect of morphine in the place preference paradigm (both sexes).	Dronabinol on P35–49 (5 and 10 mg/kg, i.p.) Suppressed sensitivity to morphine conditioning (both sexes).
[119]	SD rats (both sexes)	MD on P9 Aggressive behavior (females). Increased NMDAr density and decreased D2r density in the caudate–putamen complex (females).	THC on P37–47) (2.5, 5, and 10 mg/kg, i.p.) Impaired performance in the social recognition test (females). Increased immobility in the FST (females).	THC on P37–47 (2.5, 5 and 10 mg/kg, i.p.) Reversed aggressive behavior (females). Counteracted the increase in NMDAr density and the reduction in D2r density in the caudate-putamen (females). Increased immobility in the FST (males). Downregulation and desensitization of CB_1_R (both sexes).
[120]	Wistar rats (both sexes)	MD on P9 Anxiogenic-like effect in the hole board and EPM (males). Reduced levels of plasma leptin (both sexes).	CP on P28–42 (0.4 mg/kg, i.p.) Disrupted PPI (females). Increased adrenocortical responsiveness to PPI (males). Reduced plasma leptin levels (males).	CP on P28–42 (0.4 mg/kg, i.p.) No effects reported.
[100]	Wistar rats (both sexes)	MD on P9 Decreased CB_1_R density in the dentate gyrus and CA1 (males). Increased GFAP+ cells in the dentate gyrus (males).	CP on P28–42 (0.4 mg/kg, i.p.) Decreased CB_1_R density in the dentate gyrus and CA1 (males). Increased GFAP+ cells in the dentate gyrus (males). Reduced BDNF expression in the CA1 and CA3 (females).	CP on P28–42 (0.4 mg/kg, i.p.) Reversed the decrease in CB_1_R density in the dentate gyrus and CA1 and the increase in the GFAP+ cells (males). Increased BDNF expression in the dentate gyrus (females).
[43]	SD rats (male)	LB on P7–14 Impaired spatial recognition and social recognition memory (male).	WIN on P30–45 (1.2 mg/kg, i.p.) No effects reported.	WIN on P30–45 (1.2 mg/kg, i.p.) Impaired spatial recognition and social recognition memory (male). Less anxiety-like behavior in the open field test (male).
[122]	SD rats (both sexes)	LB on P7–14 Impaired social preference and social recognition (both sexes). Increased immobility in the FST (both sexes). Anxiety-like behavior in the open field test (both sexes). Downregulation of CB_1_R in the PFC and CA1, and GRs in the PFC and BLA (males). Downregulation of GRs in the CA1 and BLA, and CB_1_R in the BLA (females).	URB (0.4 mg/kg, i.p.) on P30–45 Impaired social preference and social recognition (both sexes). Increased immobility in the FST (both sexes). Downregulated CB1r in the PFC and CA1, and GRs in the PFC and BLA (males). Downregulation of GRs in the CA1, and CB_1_R in the BLA (females).	URB (0.4 mg/kg, i.p.) on P30–45 No effects reported.

AEA: anandamide; BDNF: brain-derived neurotrophic factor; BLA: basolateral amygdala; CB_1_R: CB1 receptor type 1; CP: CP-55,940; D2: dopaminergic receptor type 2; ELS: early life stress; EPM: elevated-plus maze; FAAH: fatty acid amide hydrolase; FST: forced swim test; GFAP+: glial fibrillary acidic protein-positive; GRs: glucocorticoid receptors; LB: limited bedding paradigm; LPS: lipopolysaccharide; MD: maternal deprivation; MS: maternal separation; NAA: N-acetyl-aspartate; NMDAr: NMDA receptors; P: postnatal day; PFC: prefrontal cortex; PPI: prepulse inhibition; SD: Sprague–Dawley; THC: delta-9-tetrahydrocannabinol; URB: URB597; WIN: WIN55,212-2.

**Table 2:** Summary of rodent model studies of exposure to cannabinoids during late adolescence.

Reference	Animals	ELS Effects	Cannabinoid Effects	Interaction Effects
[43]	SD rats (both sexes)	LB on P7–14 Impaired short-term memory, spatial location, and social recognition (both sexes). Impaired novel object recognition (males). Upregulation in PFC-GRs and downregulation in BLA-CB_1_R (males). Upregulation in PFC-GRs and CA1-CB_1_R (females).	WIN on P45–60 (1.2 mg/kg, i.p.) No effects reported.	WIN on P45–60 (1.2 mg/kg, i.p.) Prevented the ELS-induced behavioral deficits and reduced anxiety levels (both sexes). Normalized the ELS-induced upregulation in PFC-GR and downregulation in BLA-CB_1_R (males). Normalized the ELS-induced upregulation in PFC-GRs and CA1-CB_1_R (females).
[123]	SD rats (both sexes)	LB on P7–14 Impaired social preference and social recognition (both sexes). Increased immobility in the FST (both sexes). Reduced activity of the enzyme MAGL in the ventral subiculum and reduced LTP in the ventral subiculum–nucleus accumbens pathway (both sexes). Reduced BDNF expression in the nucleus accumbens and ventral subiculum (males). Increased BDNF expression in the ventral subiculum, reduced MAGL activity in the nucleus accumbens (females).	URB on P45–60 (0.4 mg/kg, i.p.) and JZL on P45–60 (2 mg/kg, i.p.) No effects reported.	URB on P45–60 (0.4 mg/kg, i.p.) and JZL on P45–60 (2 mg/kg, i.p.) Improved social preference and social recognition, reduced passive coping in the FST (both sexes). URB on P45–60 (0.4 mg/kg, i.p.) Normalized BDNF expression in the ventral subiculum (both sexes). JZL on P45–60 (2 mg/kg, i.p.) Normalized BDNF expression in the ventral subiculum (males). Normalized MAGL activity in the nucleus accumbens (females).

BDNF: brain derived neurotrophic factor; BLA: basolateral amygdala; CB_1_R: CB1 receptors type 1; ELS: early life stress; FST: forced swim test; GR: glucocorticoids; JZL: JZL184; LB: limited bedding paradigm; LTP: long-term potentiation; MAGL: monoacylglycerol lipase; P: postnatal day; PFC: prefrontal cortex; URB: URB597; SD: Sprague–Dawley; WIN: WIN55,212-2.

## Data Availability

Data sharing not applicable.
